# Imaging of Substantia Nigra in Parkinson’s Disease: A Narrative Review

**DOI:** 10.3390/brainsci11060769

**Published:** 2021-06-09

**Authors:** Paola Feraco, Cesare Gagliardo, Giuseppe La Tona, Eleonora Bruno, Costanza D’angelo, Maurizio Marrale, Anna Del Poggio, Maria Chiara Malaguti, Laura Geraci, Roberta Baschi, Benedetto Petralia, Massimo Midiri, Roberto Monastero

**Affiliations:** 1 Department of Experimental, Diagnostic and Specialty Medicine (DIMES), University of Bologna, Via S. Giacomo 14, 40138 Bologna, Italy; paola.feraco@apss.tn.it; 2 Neuroradiology Unit, S. Chiara Hospital, 38122 Trento, Italy; benedetto.petralia@apss.tn.it; 3 Section of Radiological Sciences, Department of Biomedicine, Neurosciences & Advanced Diagnostics, School of Medicine, University of Palermo, 90127 Palermo, Italy; giuseppe.latona@unipa.it (G.L.T.); elebru.91@gmail.com (E.B.); costanza.dangelo@gmail.com (C.D.); massimo.midiri@unipa.it (M.M.); 4 Department of Physics and Chemistry, University of Palermo, 90128 Palermo, Italy; maurizio.marrale@unipa.it; 5 Department of Neuroradiology and CERMAC, San Raffaele Scientific Institute, San Raffaele Vita-Salute University, 20132 Milan, Italy; delpoggio.anna@hsr.it; 6 Neurology Unit, S. Chiara Hospital, 38122 Trento, Italy; mariachiara.malaguti@apss.tn.it; 7 Diagnostic and Interventional Neuroradiology Unit, A.R.N.A.S. Civico-Di Cristina-Benfratelli, 90127 Palermo, Italy; laura.geraci@inwind.it; 8 Section of Neurology, Department of Biomedicine, Neurosciences & Advanced Diagnostics, School of Medicine, University of Palermo, 90127 Palermo, Italy; roberta.baschi@gmail.com (R.B.); roberto.monastero@unipa.it (R.M.)

**Keywords:** magnetic resonance imaging, neuromelanin, nigrosome-1, iron, biomarkers, radiomics, neurodegenerative diseases, Parkinson’s disease, parkinsonian disorders

## Abstract

Parkinson’s disease (PD) is a progressive neurodegenerative disorder, characterized by motor and non-motor symptoms due to the degeneration of the pars compacta of the substantia nigra (SNc) with dopaminergic denervation of the striatum. Although the diagnosis of PD is principally based on a clinical assessment, great efforts have been expended over the past two decades to evaluate reliable biomarkers for PD. Among these biomarkers, magnetic resonance imaging (MRI)-based biomarkers may play a key role. Conventional MRI sequences are considered by many in the field to have low sensitivity, while advanced pulse sequences and ultra-high-field MRI techniques have brought many advantages, particularly regarding the study of brainstem and subcortical structures. Nowadays, nigrosome imaging, neuromelanine-sensitive sequences, iron-sensitive sequences, and advanced diffusion weighted imaging techniques afford new insights to the non-invasive study of the SNc. The use of these imaging methods, alone or in combination, may also help to discriminate PD patients from control patients, in addition to discriminating atypical parkinsonian syndromes (PS). A total of 92 articles were identified from an extensive review of the literature on PubMed in order to ascertain the-state-of-the-art of MRI techniques, as applied to the study of SNc in PD patients, as well as their potential future applications as imaging biomarkers of disease. Whilst none of these MRI-imaging biomarkers could be successfully validated for routine clinical practice, in achieving high levels of accuracy and reproducibility in the diagnosis of PD, a multimodal MRI-PD protocol may assist neuroradiologists and clinicians in the early and differential diagnosis of a wide spectrum of neurodegenerative disorders.

## 1. Introduction

Parkinson’s disease (PD) is a progressive neurodegenerative disease that is characterized by motor and non-motor symptoms. The disease is mostly sporadic, and it is caused by the interplay between genetic and environmental factors [1]. The neuropathology of PD is characterized by neuronal degeneration in the pars compacta of the substantia nigra (SNc) with dopaminergic denervation of the striatum. Subsequently, this neuronal loss is also seen in other brain regions and non-dopaminergic neurons, with multisite involvement of the central, peripheral, and autonomic nervous system [2]. The histological hallmark of PD are Lewy bodies, which are cytoplasmic inclusions resulting from an abnormal deposition of α-synuclein aggregates. The latter are not specific to PD, but they characterize other Parkinsonisms, such as Lewy body dementia and multiple system atrophy. It is currently unknown how Lewy bodies are related to the progression of PD, and current knowledge suggests that neuronal degeneration occurs due to several processes, including neuroinflammation, oxidative stress abnormalities, mitochondrial dysfunction, and abnormalities of protein quality control [3].

According to the *gut-to-brain* transmission model of PD pathology proposed by Braak et al., changes in brainstem and subcortical structures are more evident in early disease stages, while cortical structures are principally involved in advanced-stage PD [4]. Clinical manifestations of PD primarily include bradykinesia plus at least one of resting tremor and rigidity. Supportive criteria for a PD diagnosis are a beneficial response to dopamine therapy, the presence of medication-induced dyskinesia, and (early) olfactory dysfunction [5]. Motor symptoms progressively worsen with age, leading to near total immobility in advanced-stage PD. Although PD has been primarily identified as a movement disorder, non-motor symptoms (such as hyposmia, autonomic dysfunction, mood and sleep disorders, and cognitive impairment) are very common features of the disease, and they have been associated with a poor quality of life [6]. Specifically, cognitive impairment—which encompasses a spectrum varying from mild cognitive impairment to dementia [7,8]—has been associated with poor outcomes and mortality [9].

The diagnosis of PD is to date based on clinical features, with motor symptoms constituting the core criteria [1,5]. Over the past two decades, great efforts have been invested in evaluating reliable biomarkers for PD; none of these parameters, however, have been successfully validated for routine clinical practice [10]. Among these biomarkers, magnetic resonance imaging (MRI)-based biomarkers have undoubtedly contributed to the differential diagnosis between degenerative from secondary Parkinsonism [11].

Although the sensitivity of conventional MRI sequences (i.e., T2 or T1 weighted) has been considered as *poor*, particularly in early PD, the advent of high- and ultra-high-field MRI techniques has brought many advantages to the study of brainstem and subcortical structures [11]. The distinction between PD and atypical parkinsonian syndromes (PS) (including multiple system atrophy (MSA), progressive supranuclear palsy (PSP), corticobasal syndrome (CBS), and dementia with Lewy bodies (DLB)) is challenging to establish, particularly in the early stages. However, diagnostic accuracy is important in predicting a response to levodopa or anticholinergic therapy. In addition, whilst many studies have described the MRI features of PS (especially regarding PD and MSA subtype P), it is not easy to distinguish these diseases with routine MRI. Recently, improvements in MRI technology have made possible the study of changes within the SNc, which is particularly vulnerable to degeneration in PD [12]. The SNc, which is subdivided into nigrosomes and the nigral matrix, plays an essential role in regulating movements, with classic PD motor symptoms appearing when 30% or more of its dopaminergic neurons have vanished [13,14]. Recent efforts have been focused on the development of MRI sequences in order to enhance the characterization of SNc damage in PD. These efforts regard nigrosome imaging, neuromelanin-sensitive sequences, iron-sensitive sequences, and advanced diffusion imaging [11,13,15,16]. The use of these imaging methods, alone or in combination, is emerging as an encouraging early diagnostic biomarker of PD [17]. These techniques may help to discriminate PD patients from control patients or to discriminate PD patients from atypical PS. Whilst these imaging methods are not in common use and they require specific training to achieve high levels of accuracy and reproducibility [18], their inclusion in a multimodal MRI-PD protocol may assist clinicians and neuroradiologists an arriving at a differential diagnosis. The purpose of this narrative review is to evaluate the state- of-the-art of MRI techniques, as applied to the study of SN, and their potential future applications regarding the diagnosis and treatment of PD.

## 2. Materials and Methods

**Figure 1:**
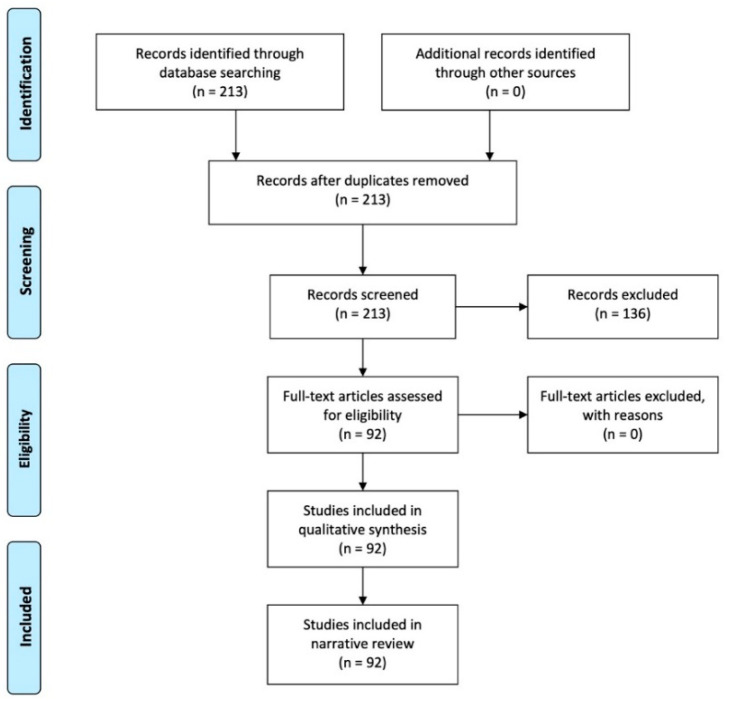
PRISMA flow diagram of studies selection.

Extensive research in English was performed in January 2021 on the literature contained in PubMed (https://pubmed.ncbi.nlm.nih.gov, accessed on 10 January 2021), using the following keywords and their combinations: *Parkinsonisms*, *Parkinson’s disease, magnetic resonance imaging, sustantia nigra, neuromelanin imaging, iron imaging, nigrosome imaging*, and *diffusion weighted and/or diffusion tensor imaging.* Preclinical and clinical studies from the last six years (January 2015–December 2020) were meticulously reviewed, focusing on new MRI sequences applied to PD. The publication date was restricted to the last six years to facilitate detailed comprehension regarding future perspectives of MRI in the study of SN in PD. Various relevant articles with this time range were included in order to maximize the topic coverage of this review. Where available, full texts in English were included, together with the most significant corresponding references. The exclusion criteria were unavailability of full text; non-English publications; case reports; reviews; and publications unrelated to PD, PS, or SN. Thereafter, the results were assessed according to the PRISMA statement (Figure 1). Recent MRI applications in PD were described from the included studies, and they were systematically organized and grouped according to a particular field of study and perspectives.

## 3. Results

Two-hundred and thirteen articles were identified from the PubMed literature search. These were subsequently screened for relevance: 136 studies were excluded according to the exclusion criteria, while 92 were included. The full text was available for all of the 92 included studies, which were included in the qualitative analysis. Considering the different study techniques, we identified the following: 20 articles relating to neuromelanin, 23 regarding nigrosome-1 imaging, 22 discussing iron imaging, 16 relating to diffusion-weighted imaging, and 11 articles referring to radiomics.

## 4. Discussion

Various neuroimaging techniques (structural and functional) have been applied to Parkinsonism over the past two decades, each providing specific information regarding underlying brain disorders [11]. Specifically, MRI has been used as a tool with which to improve diagnostic accuracy in characterizing patients with extrapyramidal symptoms. Recent efforts have focused on the development of more precise and performing MRI sequences in order to obtain an enhanced characterization of the SNc damage in Parkinsonism. These efforts include nigrosome imaging, neuromelanin-sensitive sequences, iron-sensitive sequences, and advanced diffusion imaging [11,13,15,16]. The use of these imaging methods, alone or in combination, is emerging as an encouraging early diagnostic biomarker of PD. Recent and forthcoming applications of MRI have been summarized from the available literature and grouped by *field/s of application* for this review.

### 4.1. Neuromelanin Imaging

Neuromelanin (NM) is a black pigment that is composed of melanin, proteins, lipids, and metal ions, and it is found in the SNc (in the nigral matrix and the nigrosomes). NM plays a protective role against the accumulation of toxic catecholamine derivatives and oxidative stress [19]. NM normally accumulates during aging but is strongly reduced in patients with PD as a result of the selective loss of dopaminergic neurons containing NM. The latter has a paramagnetic T1 reduction effect on MRI due to the presence of melanin-iron complexes [20]. With high-resolution turbo spin echo (TSE) T1W images with a magnetization transfer (MT) pulse, it is possible to suppress brain tissue signals due to the prolongation of the T1 relaxation time [21]. Hence, nuclei-containing NM can be visualized as a separate hyperintense area relative to the surrounding hypointense brain tissue. Although the use of TSE T1W images has been consistently applied to visualizing NM, the gradient recalled echo (GRE) sequence with MT pulse has recently been demonstrated to achieve the sharpest contrast and lowest variability when compared with a T1W TSE-MT sequence [22].

**Figure 2:**
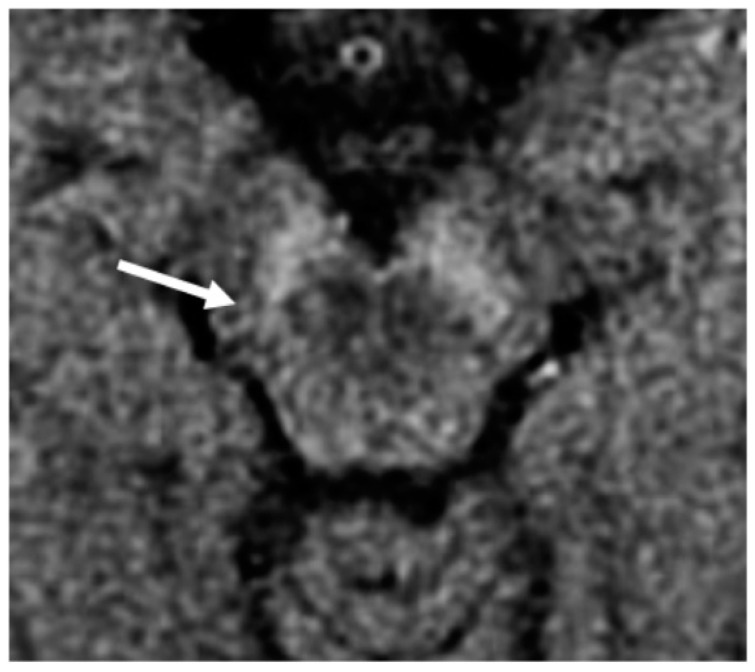
NM-MRI sequence with an explicit MT preparation pulse, scanned with a 1.5T MR scanner at the level of the SN in a PD patient with asymmetrical motor symptoms onset: the loss of hyperintensity in the posterolateral aspect of the right SN (arrow) correlated well with the clinical presentation.

NM-MRI is a validated technique with which to quantify the loss of dopaminergic neurons in the SN of patients with PD. The loss of SN hyperintensity in the T1W-MT sequence is associated with the loss of neuromelanin-containing neurons in PD and DLB, as confirmed in post-mortem studies [23]. Indeed, patients suffering from PD have significantly reduced NM signal in the SN (Figure 2), which invariably decreases on follow-up [24,25,26,27,28,29].

Measuring NM-sensitive images correlates with elevated diagnostic accuracy for PD: the sensitivity and specificity of this technique to distinguish between PD and control patients are 88% and 80%, respectively [30]. The NM signal changes commence in the posterolateral motor areas of the SN, and then proceed to the medial areas [31]. Hence, the evaluation of longitudinal changes in the NM signal in PD patients could be used as a marker indicating disease progression. A reduction in NM signal has been reported to be not specific for motor or non-motor PD subtypes [32]. On the other hand, a potential diagnostic value of NM-MRI in discriminating PD motor phenotypes has been proposed [33]. Indeed, patients with postural instability gait difficulty phenotype display increased severe signal attenuation in the medial part of the SNc, in comparison with tremor-dominant PD patients [33]. Furthermore, the use of NM-MRI-based imaging is capable of differentiating between untreated essential tremor (ET) and de novo PD with a tremor-dominant phenotype [34]. Finally, a NM signal decrease has been observed in patients suffering from idiopathic rapid eye movement sleep behavior disorder, which is considered a prodromal phase of Parkinsonism and PD [35,36].

### 4.2. Nigrosome-1 Imaging

**Figure 3:**
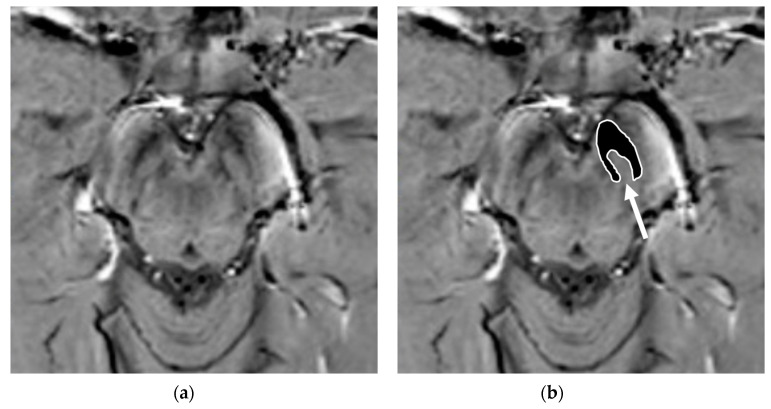
Susceptibility-weighted imaging (SWI) scan performed with a 3T MR scanner in a normally aging brain of a 65-year-old male who underwent a brain MRI examination for persistent headaches. A raw slice passing through the mesencephalon (**a**) and the same slice with superimposed highlighted SNc (white surrounded black ROI), thereby demonstrating the normal appearance of the nigrosome-1 (hyperintense area pointed by the white arrow) or swallow-tail sign (**b**).

Nigrosomes are dopaminergic neurons within the SNc that are characterized by high NM levels and a paucity of iron. They can be subdivided into five different regions (nigrosome 1 to 5), the largest of which, nigrosome-1 (located in the dorsolateral part of SNc [12]), has been shown to play a key role in the neuropathology of PD. Indeed, the greatest loss of dopaminergic neurons in PD patients occurs in the nigrosome-1. It was first detected in vivo by 7.0-Tesla (7T) MRI as a hyperintense, ovoid area on T2*-weighted images, within the dorsolateral border of the hypointense SN pars compacta [37,38]. Similar findings can be found with the more commonly used 3-Tesla (3T) MRI [39]. By using T2* or susceptibility-weighted imaging (SWI), researchers have also termed this region *dorsolateral nigral hyperintensity* or a *swallow-tail sign* (STS) (Figure 3).

**Figure 4:**
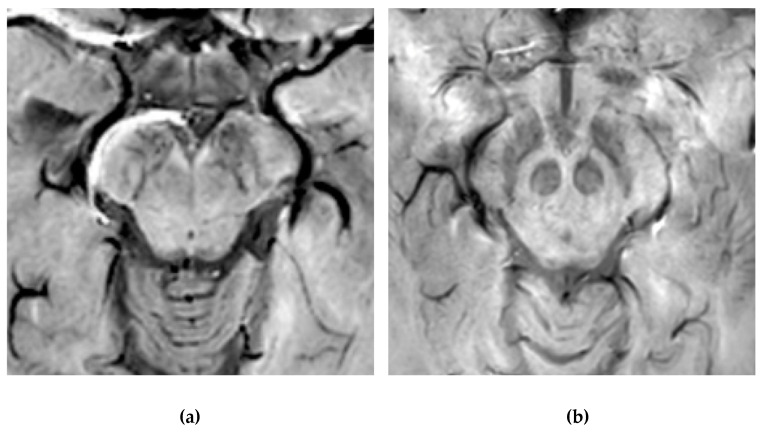
Susceptibility-weighted imaging (SWI) scan performed with a 3T MR. (**a**) Presence of regular swallow tail sign in a healthy patient; (**b**) loss of swallow tail sign in a patient with Parkinson’s disease.

Normal nigrosome-1 and the surrounding structure of the dorsolateral SN appear as a swallow tail [40], and they can be visualized in 95% of healthy subjects [41,42]. Iron deposits and microvessels have been reported as contributing to the hyposignal surrounding nigrosome-1 in the SWI of normal aged midbrains [43]. Nigrosome-1 in PD patients displays a significant loss of STS on T2* weighted images, probably due to a reduction in NM within dopaminergic neurons, an increase in free iron (which induces local inhomogeneity in the magnetic field resulting in signal loss), or a loss of paramagnetic NM–iron complexes [44,45]. As the disease advances, a loss of T2* hyperintensity in PD has been demonstrated to progress from nigrosome-1 to nigrosome-4 [46]. The absence of STS may assist in the differential diagnosis for PD if compared with controls and ET, ultimately reaching high sensitivity and specificity [17,40,47,48] (Figure 4).

Moreover, the imaging of nigrosome-1 with 3T MR has been demonstrated to differentiate drug-induced Parkinsonism from idiopathic PD with elevated accuracy, thereby being of assistance in screening patients who required dopamine transporter imaging [49]. Furthermore, a loss of STS has also been observed in patients suffering from idiopathic rapid eye movement sleep behavior disorder and DLB [50,51]. Whilst the loss of nigrosome-1 on SWI sequences may assist as a potential imaging biomarker in the diagnosis of degenerative parkinsonian syndromes, it cannot differentiate between idiopathic PD and PS [52,53]. Nevertheless, it has been reported that anatomical changes of SN, detected via the SWI sequence at 7T, may distinguish MSA and PSP from CBD [54], thereby confirming the pathological heterogeneity of these diseases. Of note, nigrosome-1 has also been visualized on 3D FLAIR images as an hyperintense structure within otherwise surrounding hypointense dorsolateral SN. Its loss can be used to predict presynaptic dopaminergic function and to diagnose PD with a high degree of accuracy [55].

Recently, it has been reported that the combined visual analysis of SN (by using NM-MRI and nigrosome-1 imaging, displaying normal NM in SNc and nigrosome-1 loss) has enabled the distinction of MSA-P from PD and healthy controls [56]. Moreover, it has also been described that a stratification of the swallow tail sign, using a scale on SWI-map imaging, may serve as a useful imaging biomarker regarding the differential diagnosis of Parkinsonism [57]. However, the veracity of these results must be confirmed by larger cohort studies.

### 4.3. Iron Imaging

Together with a degeneration of dopaminergic neurons, iron overload has been implicated in the pathology and pathogenesis of PD and PS. Iron deposition initially occurs in SN; however, abnormal iron levels have also been detected in the basal ganglia, thalamus, and cortex of PD patients [58].

**Figure 5:**
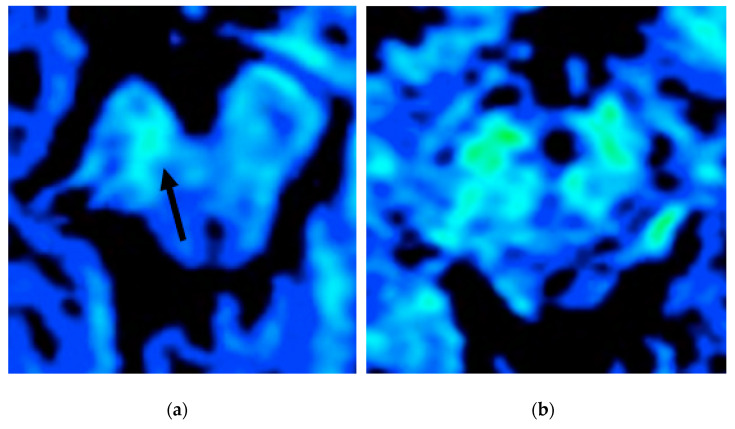
T2* map study (color scale) of two patients with PD in an evaluation of iron deposition within the SN (blue: less iron deposition; green: more iron deposition). The patient in (**a**) has a more evident asymmetrical iron deposition when compared to the patient in (**b**).

With the introduction of MRI, the in vivo characterization of brain iron content has become possible. The possibility of quantifying regional brain iron overload may provide more knowledge regarding the correlation between iron accumulation and parkinsonian symptoms. Indeed, extensive data have emphasized the importance of SN iron increase in PD patients compared to controls [30,35,59]. From a technical perspective, the iron content can be assessed by evaluating T2 and T2* relaxation rates, using either magnitude (R2*) or phase (quantitative susceptibility mapping, QSM) imaging. Among these methods, R2 and R2* relaxometry (i.e.,: 1/T2*, proton transverse relaxation rate which reflects increased tissue iron content) considers heterogeneities from local and adjacent tissue as being more susceptible to influence from disturbances due to calcification, micro bleeds, and myelinated fibers [11]. The R2* values in the SNc have been reported to be significantly higher in de novo PD patients with a gradual increase, which is related to disease progression [60,61] (Figure 5).

Since correlations between motor symptoms and high levels of R2* values within the SNc have been reported in PD, and R2* changes rapidly with disease progression, these methods can also be used in the prospective evaluation of PD patients [60,61]. Moreover, it has been reported that PD patients with early gait freezing pattern will have higher iron content, as evaluated by means of R2* relaxometry in the SNc, in comparison to those who do not [62].

Furthermore, QSM provides a direct measure of the local heterogeneities of the magnetic field by using a deconvolution method, which assists in eliminating the susceptibilities of surrounding structures [63]. It has been demonstrated that QSM is more sensitive than R2* in identifying iron overload in PD [63,64,65], even in the prodromal stage of the disease [66]. Values from QSM correlate with disease condition and duration [64,65,67,68], and they distinguish PD and PS [69]. Moreover, QSM can address iron variation within the SN [70] and lateral asymmetry of iron deposition, which is related to a manifestation of asymmetric signs and symptoms in PD [24]. When QSM is used in early- and advanced-stage PD patients, it is of note that it has been demonstrated that iron deposition affected SNc exclusively in the early stages of the disease, while in the late PD stage, iron deposition involved other regions, concomitant with SNc [71]. This latter finding indicates that QSM is a tool with which to monitor iron deposition and disease progression in PD. Specifically, changes in iron seem to be limited to the ventral aspect of SN [70], which has been reported to degenerate early in the course of the disease [72]. According to the distribution of the pathological involvement distinguishing the various forms of Parkinsonism, red and subtalamic nuclei are involved in PSP, together with SN, while iron deposition in MSA is significantly higher in the putamen [73]. Finally, all Parkinsonisms have been demonstrated to display increased susceptibility in the subcortical structures, thereby reflecting distinct topographical patterns of abnormal brain iron accumulation [74].

Both QSM and R2* may be effective tools in the differential diagnoses of degenerative PS, a fact that permits the tracking of dynamic changes that are associated with the pathological progression of these disorders. In addition, while QSM is more sensitive to the iron content of SN, R2* can be said to reflect pathological features, such as α-synuclein, in addition to iron deposits [75].

### 4.4. Advanced Diffusion Weighted Imaging Techniques

The loss of dopaminergic neurons in the SNc in the midbrain of patients with PD, as well as related nigral changes, are useful in differentiating neurodegenerative Parkinsonism from ET and other non-degenerative PS [64]. Routine conventional brain MRI, with an assessment of T1, T2, FLAIR, and proton density weighted sequences, is usually normal in early PD, while several studies have shown that advanced diffusion weighted imaging (diffusion tensor imaging, DTI) can assist in the early diagnosis of the disease [76]. The SN can be most clearly depicted when the diffusion gradient is applied in a left–right direction, thereby providing sharp contrast between the SN and the surrounding white matter. By depicting the white matter around SN as an area of high signal intensity, diffusion weighted imaging (DWI) reveals SN as a crescent-shaped area of low signal intensity between the tegmentum of the midbrain and the cerebral peduncle [77]. Several DTI studies have described early within-SN changes of PD patients, as compared to controls, and characterized by low fractional anisotropy (FA) values [78,79,80,81]. High resolution DTI in the SN can be useful in the diagnosis of PD, distinguish early-stage disease from controls, and has the potential to be a non-invasive early biomarker for PD diagnosis [76]. Moreover, higher SN-DTI changes have been reported to correlate with increasing dopaminergic deficits and declining α-synuclein and total tau protein concentrations in cerebrospinal fluid [80]. Furthermore, a nigral diffusion measure has been proposed as a measure of disease progression [81].

Whilst several authors have evaluated the application of DTI to studying SN in PD in the last 10 years, the results of these studies are conflicting [78,79,80,81,82,83]. For example, in their systematic review and meta-analysis, Hirata et al. estimated the mean change in SN-FA induced by PD and related diagnostic accuracy, and they concluded that SN-FA cannot be used as an isolated measure with which to diagnose PD since it displayed low sensitivity and specificity [83]. These discordant results have been hypothesized to be due to variable approaches used to demarcate the SN or unpredictable contamination of DTI evaluations from extracellular water compound or free water (FW). Hence, a FW mapping was developed, permitting the separation of the contribution of FW to DTI assessments (FW-corrected DTI) [84]. Using this approach, FW levels were observed to have increased in the posterior SN of PD patients, if compared to healthy controls, with a progressive increase during the progression of the disease. Moreover, the FW predicted the future changes in bradykinesia and cognitive status in a 1-year period, thereby providing a potential non-invasive progression marker of SN [84,85,86,87].

In addition to early PD, the FW in the posterior part of the SN has been reported to also have been increased in early MSA and PSP, as demonstrated by Arribarat et al. [88]. It has also been correlated with striatal dopaminergic denervation, thus reflecting motor and cognitive deficits. Compared PD and control patients, Planetta et al. observed an FW increase in the SN, in addition to the subthalamic nucleus, red nucleus, pedunculopontine nuclei, cerebellum, and basal ganglia in patients with PSP and MSA [86]. Several studies have demonstrated changes in water diffusivity in the SN (measured as a reduction in FA) in patients with MSA and a predominance of parkinsonian symptoms; this permits the differentiation with PD even in its early stages, when a volumetric reduction or signal change on conventional MRI are still absent [89].

There are other anatomical regions in PS (PSP, CBS, and MSA-C) that reveal microstructural anomalies, as detected by reduced FA and an increased MD. Studying changes in SN is, therefore, not indicated regarding a differential diagnosis of atypical Parkinsonism. For example, abnormal DTI in the cerebellum and MCP seems to be mainly involved in MSA-C; the DTI of SCP is mostly vulnerable in PSP. Abnormal DTI in supratentorial white matter regions appears to be mainly involved in CBS [90].

### 4.5. Radiomics, Artificial Intelligence, and Future Perspectives

Nowadays, an interest in NM-dedicated imaging and iron content quantification by means of artificial intelligence tools has only increased. A radiomic approach can be adopted to extract and analyze quantitative imaging features from medical images in garnering information to lend support to clinical decision making. These features are commonly correlated with patients’ clinical data by advanced computational methods, including machine and deep learning algorithms; the latter are ever more frequently used to aid in the early or differential diagnosis of PD [91,92,93,94].

Machine learning techniques are typically based on an analysis of training data (i.e., features extracted from images) and the transformation of these features into class labels. The aim of this is to develop a model that is capable of classification, prediction, and the estimation of a situation from selected known data (e.g., images) [95]. Also known as *deep neural learning* or *deep neural network*, deep learning is a subfield of machine learning, the aim of which is to “imitate” the human brain in processing data and decision making. Deep learning permits the differential interpretation of data by means of the use of different layers in the network: each network defines specific features of the data in a hierarchical system [95], and data representation is performed in conjunction with prediction (obtained via classification or regression).

Various reports have described PD diagnoses by means of machine learning techniques, such as a support vector machine algorithm, as applied to DTI, and a voxel-based morphometry of the whole brain [96,97,98]. Recently, deep neural networks have shown great promise when creating markers for PD prognosis and diagnosis by adopting convolutional neural network (CNN) regarding NM-MRI acquisitions. This algorithm automatically segments the SN region; computes class activation maps for patient classification; and, therefore, acts as a computer-aided PD diagnostic framework, using the NM signal [92]. Using CNNs to create prognostic and diagnostic biomarkers of PD from NM-MRI, Shinde et al. demonstrated the higher performance of this method when compared to a radiomics classifier, discriminating PD from PS with an accuracy of 85.7%. They also demonstrated that the left SNc plays a key role in this classification, as compared to the right SNc [92].

Another application of SN segmentation via CNN has been reported by Krupička et al. [99]. Artificial neural networks were also used to validate a dynamic, atlas-based segmentation process of the SN and to quantify NM-rich brainstem structures in PD [100]. Moreover, the application of texture analysis, by means of QSM, has been reported to successfully distinguish PD from healthy control patients, with higher performance indices, compared to R2* texture analysis [93]. This combination of radiomics and CNN features from QSM could enhance the diagnostic accuracy of PD [94]. Finally, applications of artificial intelligence tools appear to promise much since they may support the identification of radiological biomarkers in PD, and they may also reveal deeper understanding of the pathophysiological alterations in SNc.

## 5. Conclusions

MRI-based biomarkers have undoubtedly contributed to the diagnosis of PD and a differential diagnosis of PD and atypical PS over the past two decades. Improvements in MRI technology have made the study of SN microstructural changes and metal deposits possible, with both being of major importance to PD patients. An increasing number of MRI sequences and methods have been developed, resulting in more precise imaging findings that characterize SNc damage in PD. These images comprise nigrosome imaging, neuromelanin-sensitive sequences, iron-sensitive sequences, and DWI. The use of these imaging methods, alone or in combination, is emerging as an encouraging early diagnostic biomarker of PD. These techniques may also permit the discrimination of PD from control patients or PD patients from atypical PS. However, the diagnosis of PD is still based on clinical features, and these imaging methods are not yet in widespread use. Accordingly, multi-center studies deploying large cohorts are required. Results from these studies may result in the identification of new imaging biomarker of PD, thereby enabling the neuroradiologist to support clinicians in the final diagnosis of the disease.

Finally, the application of artificial intelligence tools has only increased in assisting the early or differential diagnosis of PD. A radiomic approach has also been increasingly adopted to extract and analyze quantitative imaging features from medical images, which are beyond those identifiable by an expert eye. The next step will be the inclusion of these radiomic features into the clinical decision making workflow. Such a process may also lead to extending our knowledge relating to the pathophysiological alterations of impaired brain areas, nuclei, and networks.
